# Heterogenous use of misoprostol for induction of labour: results of an online survey among midwives in German-speaking countries

**DOI:** 10.1007/s00404-021-06079-7

**Published:** 2021-05-03

**Authors:** Verena Bossung, Werner Rath, Achim Rody, Christiane Schwarz

**Affiliations:** 1 Department of Obstetrics and Gynecology, University Hospital of Schleswig-Holstein, Campus Luebeck, Ratzeburger Allee 160, 23538 Luebeck, Germany; 2 Department of Obstetrics and Gynecology, University Hospital RWTH Aachen, Pauwelsstraße 30, 52074 Aachen, Germany; 3 Department of Midwifery Science, C/O BMO, University of Luebeck, Ratzeburger Allee 160, 23562 Lübeck, Germany

**Keywords:** Induction of labour, Midwife, Misoprostol, Survey, Castor oil

## Abstract

**Purpose:**

This online survey looked at the experiences and general perceptions of midwives concerning induction of labour and the specific use of misoprostol.

**Methods:**

We published an online questionnaire with 24 questions in German on midwives’ experiences and perceptions of different methods of induction of labour.

**Results:**

The online survey was answered by 412 midwives between February 2016 and February 2017. At least 20% of the 24 questions were answered in 333 questionnaires, which were included in this analysis. Oral misoprostol was the most common induction method for primipara and for women with a previous vaginal birth and an unfavourable cervix. Apart from alternative methods for induction of labour like castor oil and complementary/alternative methods, oral misoprostol was the preferred method of induction of labour by midwives. Midwives described a wide range of dosage schedules concerning application intervals, starting doses, and the maximum daily dose of misoprostol. Approximately 50% of the participants of this study described prescriptions of more than 200 µg misoprostol daily for induction of labour.

**Conclusion:**

Misoprostol is widely used in Germany and was one of the three preferred methods of induction of labour among midwives in our study next to castor oil and complementary/alternative methods. The preparation and dosage of misoprostol vary significantly among hospitals and do not adhere to international guidelines. Midwives voiced their concerns about inconsistent indications and heterogenous use of different methods and dosages of induction. They wished for more patience with late-term pregnancies and individualized shared decision-making between pregnant women and obstetricians.

**Supplementary Information:**

The online version contains supplementary material available at 10.1007/s00404-021-06079-7.

## Introduction

Induction of labour (IOL) is an increasingly common intervention in obstetrics world-wide. In Germany, more than 22% of all deliveries are induced [1]. The most common indications for IOL include post-term pregnancy, premature rupture of membranes, fetal indications as suspected fetal macrosomia or fetal growth restriction, as well as maternal risks as preeclampsia, diabetes, and cholestasis of pregnancy. The rates of elective inductions are also rising steadily in developed countries. In recent years, numerous randomized-controlled trials (RCT) regarding the risks and benefits of IOL have been published. The results are heterogenous: In some studies, benefits as the reduction of stillbirths for low-risk pregnancies at 41 weeks [2], the reduction of shoulder dystocia for fetuses with suspected macrosomia [3], and the reduction of caesarean section rates in low-risk nulliparous women at 39 weeks have been reported [4], while in others, no reduction of caesarean deliveries or stillbirths could be found in correlation with increasing induction rates over periods of time [5–7].

There is a variety of different methods for IOL including pharmacological, mechanical, and complementary methods. The most common pharmacological agents are oxytocin, misoprostol, and dinoprostone (prostaglandin E2, PGE2) in different application routes and forms (intravenous for oxytocin, oral tablets, and vaginal tablets/gels/membranes for prostaglandins). Amniotomy, ripening of the cervix with balloon catheters (e.g., Foley), or membrane sweeping comprise the possibilities of mechanical induction. Complementary methods include castor oil orally, clove oil for vaginal use as well as other complementary and alternative methods (CAM) like homeopathy or acupuncture. The preferred methods of induction vary around the world. Although the World Health Organization (WHO) recommends prostaglandins as first-line pharmacological induction agents [8] and they are frequently used in Europe, oxytocin remains popular in many regions like Latin America, Africa, or Australia [9].

Misoprostol is a synthetic prostaglandin E1 analogue and has been extensively studied for IOL. Numerous clinical trials and meta-analyses exist on the efficacy and safety of misoprostol [10–13]. They have shown that misoprostol is more effective than vaginal PGE2 for labour induction with a lower caesarean section rate [11, 14]. For many years, misoprostol has been part of the WHO’s “Model List of Essential Medicines”, which includes the most efficient, safe, and cost–effective medicines used in health care systems world-wide [15]. It is listed for the treatment of abortion and postpartum haemorrhage. Misoprostol was first licensed in 1985 for the treatment of gastroduodenal ulcers and is contraindicated in pregnancy, as stated by its manufacturing company. In Germany, it was withdrawn from the market in 2006. Misoprostol can be purchased under the trade name “Cytotec” containing 200-µg misoprostol per tablet. In contrast to other European countries, there has been no licensed drug for IOL containing misoprostol in Germany, since “Misodel”, a licensed drug containing 200-µg slow-release misoprostol for vaginal use, was withdrawn from the market in 2019. A new medication named “Angusta”, containing 25-µg misoprostol, has only recently been licensed in Germany and was not available at the time of this survey. Apart from induction of labour, misoprostol is used for termination of pregnancy, cervical priming before surgical abortion, and the treatment of postpartum haemorrhage. Since there was no licensed medication containing misoprostol in Germany, recommendations from a national guideline regarding dosage and application intervals of misoprostol did not exist for a long time until recently [16]. As a consequence, professionals who use misoprostol for IOL in Germany have to produce their own preparations by local hospital pharmacies using the non-licensed 200-µg tablets and have to consider national or international recommendations on the use of misoprostol. The International Federation of Gynaecology and Obstetrics (FIGO) has published a dosing chart for misoprostol in its different indications [17] and the WHO published recommendations for IOL which include statements on misoprostol use [8]. The recommendations give suggestions for the route of application, the timing and dosage, as well as for the maximum daily dose. Despite extensive knowledge on misoprostol use for IOL from the international literature [10, 12, 13], detailed data on its use in Germany are sparse [18].

This study aims to explore the use of different methods of IOL in German-speaking countries and midwives’ experiences with these methods. Although physicians are indicating labour induction and determine the appropriate method, midwives are closely involved in its application and the following birth process. Our online survey focuses upon the application of misoprostol including detailed questions on dosing, preparation, and side effects.

## Materials and methods

### Questionnaire

We created an online questionnaire in German with 22 questions about different aspects of IOL. Midwives were questioned on their general experiences with different methods of IOL and on complications or side effects they may have experienced. They were specifically questioned on the use of misoprostol, the dosing schedules, and the routes of application. Finally, we collected data about the midwives’ personal work situation, their department’s annual birth numbers and level of perinatal care. The survey contained closed and open questions. The invitation to participate using an online link (barcode) to the questionnaire was distributed via professional midwife organizations, via midwifery journals and via social media.

### Respondents

German-speaking midwives were asked to answer the questions between February 2016 and February 2017. Answers were collected via www.surveymonkey.com. The participation was anonymous and voluntary.

### Statistical analysis

For statistical analysis, we included questionnaires with at least 20% of the 22 questions completed (five or more). Data was extracted as.csv files from the Surveymonkey Collector. Descriptive analysis was performed with MS Excel for Office 365 and IBM SPSS Statistics version 25. *p* values were derived from Pearson’s Chi-square test or from Fisher’s exact test, and the type I error level was set to 0.05.

## Results

### Characteristics of respondents

Between February 2016 and February 2017, the online survey was answered by 412 midwives. 333 midwives answered at least 20% of the 22 questions (five or more) and were included in this analysis. Please see Table 1 for the respondents’ characteristics. Most participants of the survey worked as midwives in a hospital only or in a hospital in combination with ante- and postnatal outpatient care. One-third of the midwives were employed by a tertiary hospital, 19.9% (*n* = 57) by subspecialty care centres. Most midwives participating in this survey worked in hospitals with 1001–2000 births per year, 30.1% (*n* = 87) in centres with 501–1000 annual births. The respondents worked in German-speaking countries, most of them in Germany (92.5%, *n* = 308). 25 midwives were employed in Switzerland or Austria (7.5%).

**Table 1:** Characteristics of respondents

Variable	Distribution	Total (*n* = 333)	%
Work situation	All answers	329	100
	Hospital only	184	55.9
	Hospital + ante/postnatal care	105	31.9
	Ante/postnatal care only	30	9.1
	Others	10	3.0
Level of hospital care	All answers	287	100
	1 (tertiary hospital)	91	31.7
	2 (subspecialty care centre)	57	19.9
	3 (specialty care)	16	5.6
	4 (basic care)	113	39.4
Births per year of hospitals	All answers	289	100
	< 500	39	13.5
	501–1000	87	30.1
	1001–2000	121	41.9
	2001–3000	32	11.1
	> 3000	10	3.5

Percentages are given as column percentages

### Methods of induction used in German-speaking countries

**Fig. 1:**
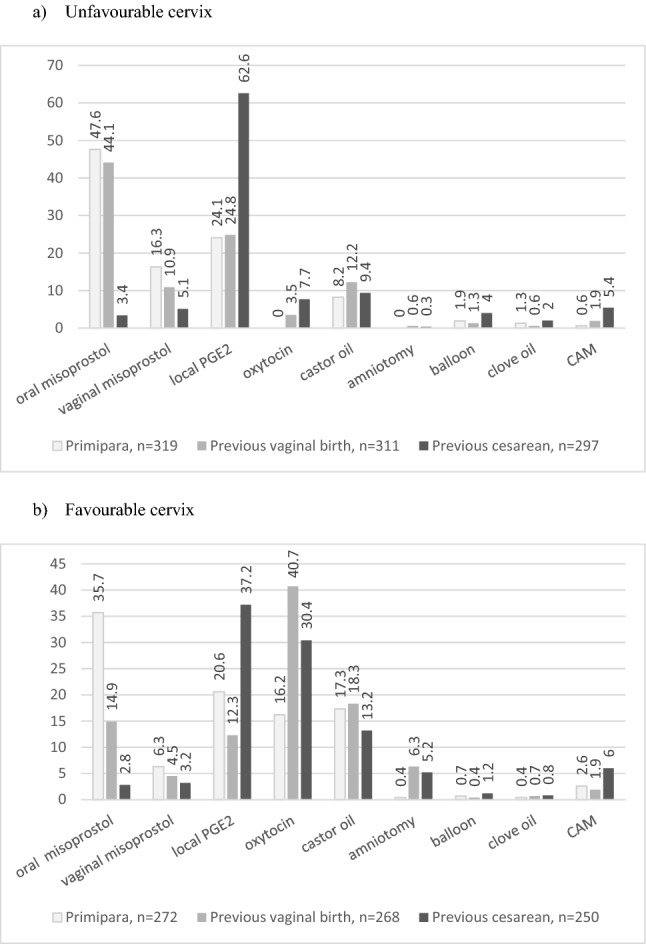
Which method of IOL is mostly used in your hospital/your region? (data given in %). **a** Unfavourable cervix. **b** Favourable cervix

The midwives were asked which methods of IOL were used in their hospital or their region. We specifically asked them about IOL in patients with an unfavourable cervix (Bishop Score < 6) versus a favourable cervix and for IOL in primiparous women versus women with a previous vaginal birth or previous caesarean section (see Fig. 1 and supplementary table 1). The most frequently used method of induction in primiparous women with a favourable or unfavourable cervix was oral misoprostol followed by vaginal or cervical PGE2. For primiparous women with an unfavourable cervix vaginal misoprostol and castor oil were also frequently used. For IOL in primiparous women with a favourable cervix castor oil and oxytocin were used by 17.3% (*n* = 47) and 16.2% (*n* = 44) of the participants. After a previous vaginal birth, oral misoprostol still maintained to be the most frequently used method of IOL in patients with an unfavourable cervix, followed by vaginal or cervical PGE2, castor oil, and vaginal misoprostol. In patients with a favourable cervix and a previous vaginal birth, IOL was mostly performed with oxytocin, castor oil, and oral misoprostol. After a previous caesarean section, most women with an unfavourable cervix were induced with vaginal or cervical PGE2, followed by castor oil, oxytocin, and CAM. For women with a favourable cervix and a previous caesarean section, the most frequently used methods for IOL were vaginal or cervical PGE2, oxytocin, and castor oil. Notably, misoprostol was used for IOL after a previous caesarean section by 8.4% (*n* = 25) for patients with an unfavourable cervix and by 6.0% (*n* = 15) for patients with a favourable cervix.

### Standard operational procedure for IOL

We asked midwives if their institution had a standard operational procedure (SOP) for IOL. 81.2% (*n* = 220) had an SOP, 13.3% (*n* = 36) did not have an SOP, and 5.5% of the midwives (*n* = 15) did not know if their obstetric unit had an SOP for labour induction. We found a statistically significant difference between the presence of an SOP and the hospitals’ level of care (*p* = 0.009). Midwives working in level 1 centres were informed of an SOP in 81.4% (*n* = 70/86), midwives in level 2 in 92.9% (*n* = 52/56), level 3 in 81.3% (*n* = 13/16), and midwives working in hospitals of basic care (level 4) only in 75.2% (*n* = 85/113).

### Experiences of midwives with IOL methods

**Fig. 2:**
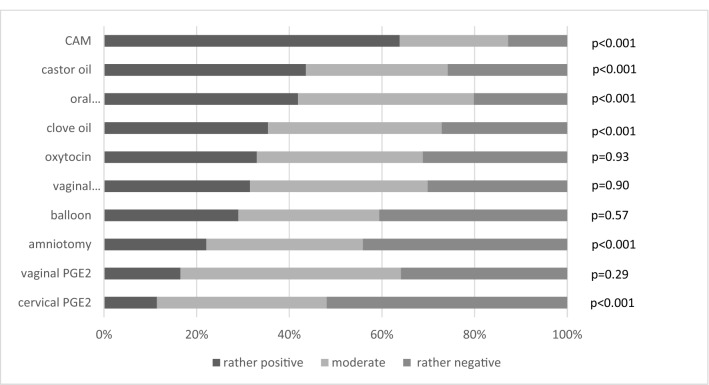
What is your general experience with the following methods of IOL?

Exploring midwives´ general perception of different induction methods, we asked if their experience with each method of IOL was rather positive, moderate, or rather negative (see Fig. 2 and supplementary table 3). CAM and castor oil were rated the most positive in our survey and there were very few reports of negative experiences with CAM. Oral misoprostol was also rated as rather positive by 41.9% (*n* = 108/258) of midwives, as moderate by 38% (*n* = 98/258) and as rather negative by 20% (*n* = 52/258). This rating was significantly better than the average rating of all IOL methods investigated (*p* < 0,001). Cervical PGE2 received the worst ratings by midwives with the lowest reported level of positive experiences.

### Side effects of different IOL methods

**Fig. 3:**
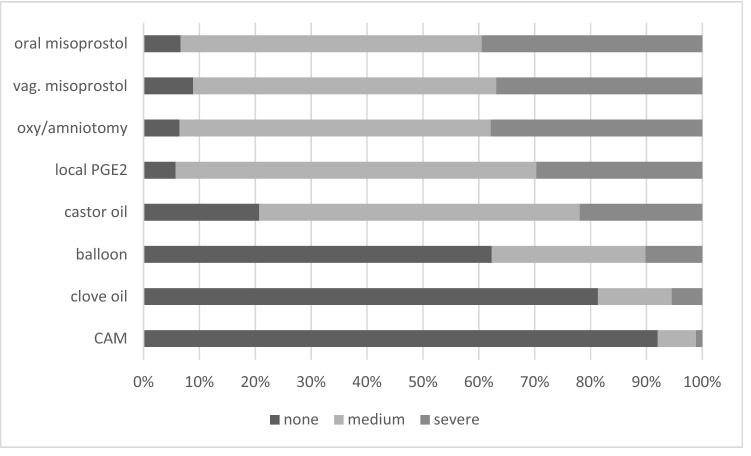
Have you experienced side effects with the following methods of IOL?

Midwives were asked to classify side effects of the different methods as severe (e.g., uterine rupture, placental abruption, severe fetal distress requiring emergency birth, or postpartum haemorrhage) or as moderate/mild (pain, uterine tachysystole, or transient fetal distress) (see Fig. 3 or supplementary table 3). They could choose their answers from a dropdown menu. It was possible to give multiple answers or to choose “no complications”. The induction methods with the best reported safety profile were CAM, clove oil, and cervical ripening balloon catheters. Oral and vaginal misoprostol, PGE2, and oxytocin combined with amniotomy were altogether associated with a significant rate of severe side effects. Castor oil was described with both severe side effects (22.1%, *n* = 54/246) and no side effects (20.7%, *n* = 51/246). Participants reported the highest rate of severe adverse outcomes with vaginal misoprostol (44.3%, *n* = 100/271).

### Midwives' preferences for IOL

**Fig. 4:**
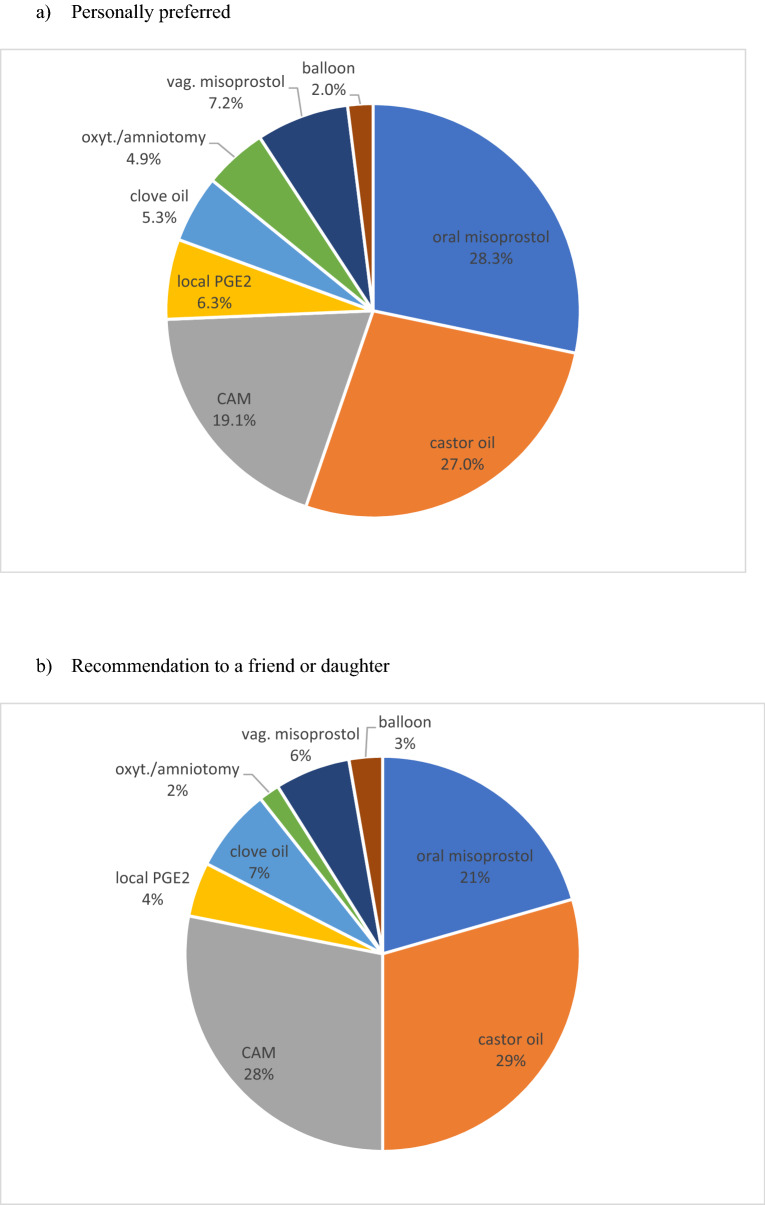
What is your personally preferred method of IOL? Which method of IOL would you recommend to a friend or your daughter? **a** Personally preferred. **b **Recommendation to a friend or daughter

The next question asked about the personal preferences of midwives (see Fig. 4 and supplementary table 4). Most midwives preferred oral misoprostol for IOL, closely followed by castor oil and CAM. None of the other methods of IOL were favoured by midwives, although some of those methods as PGE2 and oxytocin are commonly used in hospitals of German-speaking countries. We also asked midwives which method of IOL, and they would recommend to a friend or their daughter. Midwives privately preferred alternative methods of IOL as castor oil and CAM, but oral misoprostol remained popular for IOL among midwives in this question. Oxytocin/amniotomy as well as dilation of the cervix with a balloon catheter were not frequently recommended by midwives. In another question, midwives were invited to voice their views and concerns on IOL in a free text answer. 213 midwives replied and they addressed four central topics: they asked for local and nation-wide recommendations, for clear and honest communication of risks and benefits of IOL with the pregnant women during the informed consent process, they asked for more rigorous indications and for more patience from doctors and patients in the context of post-term pregnancy.

### Misoprostol: route of application, dose, and dosage intervals

We asked the midwives more specifically about the way they used misoprostol in their hospitals (see Table 2): 58.7% (*n* = 115) of the labour wards received prepared tablets in a defined dose by their local hospital pharmacy, prepared from 200-µg tablets, 39.8% (*n* = 78) divided the tablet into smaller portions directly in the labour ward, and only a few dissolved Cytotec 200 in water and gave corresponding portions of the self-prepared solution to patients.

**Table 2:** Use of misoprostol

Question	Possible answers	*n*	%
Preparation	All answers	196	100
	Divide tablets in labour ward	78	39.8
	Prepared tablets by hospital pharmacy	115	58.7
	Dissolve tablets in water	9	4.6
Route of application^a, b^	All answers	223	100
	Oral	181	81.9
	Vaginal	42	18.8
Starting dose oral^a^	All answers	180	100
	25 µg	93	51.6
	50 µg	84	46.7
	100 µg	3	1.6
Starting dose vaginal^a^	All answers	33	100
	25 µg	17	51.5
	50 µg	13	39.4
	100 µg	3	9.1
Dosing intervals oral	All answers	174	100
	2-hourly	14	8.1
	4-hourly	133	76.4
	6-hourly	18	10.3
	Variable	9	5.2
Dosing intervals vaginal	All answers	46	100
	2-hourly	0	0
	4-hourly	10	21.7
	6-hourly	33	71.7
	Variable	3	6.5
Dosing schedule oral	All answers	163	100
	Increasing dose	87	53.4
	Fixed dose	53	32.5
	Variable	23	14.1
Dosing schedule vaginal	All answers	40	100
	Increasing dose	13	32.5
	fixed dose	17	42.5
Maximum daily dose	All answers	154	100
	Up to 100 µg	15	9.7
	101–200 µg	62	40.3
	201–300 µg	41	26.6
	301–400 µg	32	20.8
	No maximum defined	4	2.6

^a^Application for primipara with IOL

^b^It was possible to choose one or both answers, *n* = 223 is the total number of answers. Percentages are given as column percentages

Most midwives/hospitals applied misoprostol orally and fewer vaginally. 87 of 242 participants stated that their hospital used “Misodel”, which was a licensed slow-release vaginal insert containing 200-µg misoprostol commercially available between 2014 and 2019.

Most obstetric units preferred a 4-hourly dosing interval for oral misoprostol and a 6-hourly interval for vaginal misoprostol. A 6-hourly dosing interval as recommended by the WHO 2011/2013 for vaginal misoprostol [8, 19] was used by none of the participants and a 2-hourly interval as recommended for oral misoprostol by 8.1% (*n* = 14). More than half of our respondents increased the oral dose during labour induction (53.4%, *n* = 87), and one-third applied a fixed dose. The starting dose mostly used for oral misoprostol was 25 µg or 50 µg. For vaginal misoprostol, most obstetric units used 25 µg or 50 µg as a starting dose (see Table 2).

Considering the maximum daily dose of misoprostol for labour induction, 50.0% of the midwives used up to 200-µg misoprostol daily and 50.0% a higher daily dose, and 20.8% of respondents (*n* = 32) used 301–400-µg misoprostol daily (see Table 2). There was no statistically significant difference in the use of less or more than 200-µg misoprostol daily between the hospitals’ different levels of care (*p* = 0.338).

## Discussion

Induction of labour is an increasingly common intervention in obstetrics. Induction rates in Western European countries range from 20 to 30%. Although obstetricians are usually indicating labour induction and determine the appropriate method, the hospitals’ midwives are closely involved in this process by taking care of the pregnant women. Therefore, we were interested in the midwives’ experiences and perceptions of different methods of IOL as well as the use of misoprostol in labour wards of German-speaking countries.

Our survey shows that oral misoprostol is widely used in German labour wards, despite the fact of not being licensed for obstetric indications in Germany at the time of our survey. International guidelines recommend the use of misoprostol for IOL and numerous clinical trials, and meta-analyses have confirmed the efficacy and safety of particularly oral misoprostol [10–13, 17, 19, 20]. It was the most preferred method of IOL for primiparous women and for women with a previous vaginal birth and an unfavourable cervix in our study. Women with a favourable cervix and a previous vaginal birth were mostly induced with oxytocin. After a previous caesarean section, the midwives reported that PGE2 was usually used for labour induction in patients with an unripe cervix and oxytocin in patients with a ripe cervix. Contrary to current guideline recommendations [8, 17, 20], misoprostol was used even in patients who had a previous caesarean section in up to 8.4% (*n* = 25). The wide-spread use of misoprostol found in our survey is in line with a nation-wide German survey published in 2013 [18]. The authors reported that 66% of German hospitals used misoprostol for IOL. Despite the off-label use, effectiveness, patients’ acceptance, cost-effectiveness, and well-proven experiences were the most common reasons for using misoprostol. Off-label use is a common problem in obstetrics globally, which may lead to medico-legal conflicts [21–24].

Our survey reveals that midwives favour oral misoprostol for IOL although most of them have experienced significant side effects. Approximately 90% of midwives in our study have observed side effects associated with the use of misoprostol, 40% (*n* = 139) of side effects were categorized as severe and 50% (*n* = 189) as moderate. This is a concerning finding in the light of a non-standardized induction regime with an unlicensed drug. Although midwives were aware of its side effects, they preferred misoprostol to PGE2 or oxytocin. Only CAM and castor oil showed a similar preference in our survey. This might be caused by the midwives’ perception that CAM (and clove oil) do not cause any side effects (see Fig. 3), which makes them a favourable option.

The most striking finding of this survey was the wide spectrum of dosing schedules for misoprostol in hospitals of German-speaking countries. We assume that this may be attributed to some uncertainties with the use of misoprostol caused by the lack of national guideline recommendations at the time of the survey. Although international guidelines from the WHO [8] or FIGO [17] as well as other countries’ national societies [20, 25] exist, they do not seem to have a significant influence on the daily induction practice in German hospitals. Only a part of the respondents obviously followed international recommendations by FIGO/WHO [8, 17] like the 2-hourly dosing interval and the use of 25-µg low-dose oral misoprostol. With respect to our data, misoprostol was mostly used orally in 4-hourly intervals and a maximum daily dose of more than 300-µg misoprostol was common (23.4%, *n* = 36). It is well documented in the literature that the rate of side effects associated with the use of misoprostol is dose-dependent. The rate of uterine hyperstimulation syndrome has been shown to increase with single doses of more than 50 µg compared to lower doses of misoprostol [20, 26]. Another crucial point is the use of small self-prepared pieces by cutting the misoprostol tablet which may lead to pharmacological inaccuracy and safety concerns. This was practiced by 40% of our respondents. The WHO has recommended to dissolve the 200-µg tablet in water [8], which was practiced by only 4.6% of our respondents. The need for self-preparation is caused by the fact that there has been no licensed drug containing misoprostol in a dose of 25 or 50 µg for obstetrical use in Germany. This is not the case in several other European countries like France or northern Europe, where “Angusta”, a drug containing 25 µg of Misoprostol and manufactured by Azanty A/S, has been licensed several years ago for IOL. The approved dosing regimens for misoprostol are either 25 µg every 2 h or 50 µg every 4 h, both orally and up to a daily maximum of 200 µg. Meanwhile, a national guideline on induction of labour has been published in December 2020 [16]. It includes general recommendations on the use of misoprostol for IOL. It states that misoprostol is the most effective agent for IOL, but patients need to be informed about its off-label use. It should be applied orally and single doses of > 50 µg (initial application) and > 100 µg (consecutive application) should be avoided. Preparations containing misoprostol should be produced by a pharmaceutical institution. “Angusta” has only recently been licensed in Germany, but cannot be purchased yet.

The use of misoprostol for IOL after a previous caesarean section reported by 8.4% of our participants represents a safety concern. Despite a lack of large RCTs [27], there are numerous reports demonstrating a significantly increased rate of uterine rupture when using misoprostol for a trial of labour after caesarean (TOLAC) [28–30]. According to all current international guidelines, the use of misoprostol for TOLAC is contraindicated [8, 17, 20, 25].

The high ratings for castor oil were a surprising result of this survey. According to our data, castor oil is commonly used for IOL in pregnant women with a favourable cervix in Germany. It was the most preferred method for induction among midwives, although almost 80% of our participants reported severe (20%, *n* = 55) or moderate (60%, *n* = 143) side effects. The use of castor oil is problematic as the method is not standardized with respect to dose, application intervals, and form of preparation. The onset of action is unpredictable. Data exist from a small RCT from 2018 comparing castor oil to placebo, reporting that more women entered into active labour after the administration of castor oil compared to placebo [31]. A 2013 review including only three RCTs (233 women) concluded that there is a lack of evidence on its efficiency and concerns on its safety profile exist [32]. Nevertheless, 60% of the midwives rated it positively for IOL and used it frequently. This may be due to castor oil being perceived harmless by midwives and pregnant women, as it is a “natural remedy” [33]. It may also be due to a lack of alternatives which could be perceived as safe and acceptable. The recently published German guideline states that castor oil should only be used in research settings [16].

The fact that more than 13% of obstetric units do not have an SOP on labour induction and the use of misoprostol, or that midwives were not aware of one, shows an urgent need for guidance and a better communication. In our survey, midwives voiced their concerns about missing local and nation-wide recommendations on indications and dosing schedules. On the national level, action has been taken. Locally, each department needs to adapt national guidance to local circumstances within an SOP. However, it is questionable if the presence of guidelines and SOPs will change the midwives’ preferences for the different methods of IOL.

There are some limitations to our study: As the survey was answered online on the platform www.surveymonkey.com by midwives who had been asked to participate by email, the sample who participated might not be representative of the average German-speaking midwife community. Possibly, several midwives from the same institution or the same region have participated in the survey, which may lead to a selection bias. Therefore, our survey cannot be representative neither for all German midwives nor for all German hospitals. Furthermore, the online survey was done from 2016 to 2017. During this period, a licensed preparation of vaginal misoprostol was available in Germany (Misodel), which has been withdrawn from the market in 2019. Based on this withdrawal, we suggest that the use of vaginal misoprostol has decreased since then. Our survey did not ask for aspects of patients’ perspectives on induction. Consistent with the variety of regimes used in hospitals, there is a fair chance that the information policy for patients may be equally heterogenous. A previous study from Germany showed that women who were induced wanted more detailed information and choice [34]. In line with that, midwives taking part in our study wished for more communication of risks and benefits of IOL with the pregnant women during the informed consent process and they asked for rigorous indications as well as for more patience from doctors and patients in the context of post-term pregnancy.

## Conclusions

The fact that more than 13% of obstetric units do not have an SOP on labour induction and the use of misoprostol, or that midwives were not aware of one, shows an urgent need for guidance and a better communication. Midwives hoped that national guidelines would lead to a standardization of dosing schedules of misoprostol and to a higher awareness of its contraindications like a previous caesarean section. For the first time, a guideline of the German obstetric societies was published in December 2020 after controversial public debates on the safety of misoprostol for labour induction in the media. Furthermore, midwives voiced their concerns about inconsistent indications and heterogenous use of different methods and dosages of induction. They would wish for more patience with late-term pregnancies and individualized shared decision-making between women and obstetricians.

## Supplementary Information

Below is the link to the electronic supplementary material.

Supplementary file1 (DOCX 20 KB)
