# Supplementary material for: Heterogenous use of misoprostol for induction of labour: results of an online survey among midwives in German-speaking countries
Source: Arch Gynecol Obstet. 2021 May 3;304(6):1501–11. doi: 10.1007/s00404-021-06079-7 (PMC8553731; doi:10.1007/s00404-021-06079-7)
Supplement: Supplementary file 1 — Supplementary file1 (DOCX 20 KB) [file 404_2021_6079_MOESM1_ESM.docx]

**Supplementary Material**

Supplementary Table 1: Which method of IOL is mostly used in your hospital/your region?

|  | Unfavourable cervix % (n) | | | Favourable cervix % (n) | | |
| --- | --- | --- | --- | --- | --- | --- |
|  | Primipara | Previous vaginal birth | Previous caesarean | Primipara | Previous vaginal birth | Previous caesarean |
|  | n=319 | n=311 | n=297 | n=272 | n=268 | n=250 |
| oral misoprostol | 47.6 (152) | 44.1 (137) | 3.4 (10) | 35.7 (97) | 14.9 (40) | 2.8 (7) |
| vaginal misoprostol | 16.3 (52) | 10.9 (34) | 5.1 (15) | 6.3 (17) | 4.5 (12) | 3.2 (8) |
| Vaginal/cervical PGE2 | 24.1 (77) | 24.8 (77) | 62.6 (186) | 20.6 (56) | 12.3 (33) | 37.2 (93) |
| oxytocin | 0 (0) | 3.5 (11) | 7.7 (23) | 16.2 (44) | 40.7 (109) | 30.4 (76) |
| castor oil | 8.2 (26) | 12.2 (38) | 9.4 (28) | 17.3 (47) | 18.3 (49) | 13.2 (33) |
| amniotomy | 0 (0) | 0.6 (2) | 0.3 (1) | 0.4 (1) | 6.3 (17) | 5.2 (13) |
| balloon catheter | 1.9 (6) | 1.3 (4) | 4 (12) | 0.7 (2) | 0.4 (1) | 1.2 (3) |
| clove oil | 1.3 (4) | 0.6 (2) | 2 (6) | 0.4 (1) | 0.7 (2) | 0.8 (2) |
| CAM | 0.6 (2) | 1.9 (6) | 5.4 (16) | 2.6 (7) | 1.9 (5) | 6 (15) |
|  |  |  |  |  |  |  |

Percentages are given as column percentages. CAM= complementary and alternative methods, PGE2= prostaglandin E2

Supplementary Table 2: What is your general experience with the following methods of IOL?

|  | Rather positive % (n) | Moderate % (n) | Rather negative % (n) | Total n | p |
| --- | --- | --- | --- | --- | --- |
| oral misoprostol | 41.9 (108) | 38.0 (98) | 20.2 (52) | 258 | <0.001 |
| vaginal misoprostol | 31.5 (23) | 38.4 (28) | 30.1 (22) | 73 | 0.933 |
| vaginal PGE2 | 16.5 (45) | 47.6 (130) | 35.9 (98) | 273 | <0.001 |
| cervical PGE2 | 11.5 (15) | 36.6 (48) | 51.9 (68) | 131 | <0.001 |
| oxytocin | 33.0 (92) | 35.8 (100) | 31.2 (87) | 279 | 0.897 |
| amniotomy | 22.1 (47) | 33.8 (72) | 44.1 (94) | 213 | <0.001 |
| castor oil | 43.6 (98) | 30.7 (69) | 25.8 (58) | 225 | 0.287 |
| Balloon catheter | 29.0 (20) | 30.4 (21) | 40.6 (28) | 69 | <0.001 |
| clove oil | 35.4 (34) | 37.5 (36) | 27.1 (26) | 96 | 0.566 |
| CAM | 63.8 (60) | 23.4 (22) | 12.8 (12) | 94 | <0.001 |

Percentages are given as row percentages. PGE2= prostaglandin E2, CAM= complementary and alternative methods. P-values compare rather positive vs. moderate vs. rather negative and are calculated with Pearson's chi-squared test.

Supplementary Table 3: Have you experienced side effects with the following methods of IOL?

|  | None % (n) | Moderate % (n) | Severe % (n) | Total % (n) |
| --- | --- | --- | --- | --- |
| oral misoprostol | 6.6 (23) | 53.9 (187) | 39.6 (137) | 100 (347) |
| vaginal. misoprostol | 8.9 (24) | 54.2 (147) | 44.3 (100) | 100 (271) |
| Vaginal/cervical PGE2 | 5.7 (29) | 64.6 (326) | 30.1 (150) | 100 (505) |
| castor oil | 20.7 (51) | 57.3 (141) | 22.1 (54) | 100 (246) |
| oxy/amniotomy | 6.4 (24) | 55.7 (209) | 38.0 (142) | 100 (375) |
| Balloon catheter | 62.3 (43) | 27.5 (19) | 12.3 (7) | 100 (69) |
| clove oil | 81.3 (74) | 13.2 (12) | 5.5 (5) | 100 (91) |
| CAM | 92.0 (81) | 6.8 (6) | 1.1 (1) | 100 (88) |
| p | <0.001 | <0.001 | <0.001 |  |

Percentages are given as row percentages. CAM= complementary and alternative methods, PGE2= prostaglandin E2

Supplementary Table 4: What is your personally preferred method of IOL? Which method of IOL would you recommend to a friend or your daughter?

|  | Personally preferred method of IOL | | Recommendation to a friend or daughter | |
| --- | --- | --- | --- | --- |
|  | n | % | n | % |
| oral misoprostol | 86 | 28.3 | 60 | 20.5 |
| castor oil | 82 | 27.0 | 86 | 29.5 |
| CAM | 58 | 19.1 | 82 | 28.1 |
| Vaginal/cervical PGE2 | 19 | 6.3 | 13 | 4.5 |
| clove oil | 16 | 5.3 | 20 | 6.8 |
| oxytocin/amniotomy | 15 | 4.9 | 5 | 1.7 |
| vaginal misoprostol | 22 | 7.2 | 18 | 6.2 |
| Balloon catheter | 6 | 2.0 | 8 | 2.7 |
| Total | 304 | 100 | 292 | 100 |

Percentages are given as column percentages. CAM= complementary and alternative methods, PGE2= prostaglandin E2
